# Effectiveness and efficiency of teleimaging in the transplantation process: a mixed method protocol

**DOI:** 10.1186/s12913-019-4488-0

**Published:** 2019-09-18

**Authors:** Kevin Zarca, Jean-Claude K. Dupont, Lorène Jacoud, Julie Bulsei, Olivier Huot, Hélène Logerot, Isabelle Durand-Zaleski

**Affiliations:** 1 AP-HP, DRCI-URC ECO, Paris, France; 2 AP-HP Hospital Henri Mondor, Creteil, France; 3 INSERM UMR 1153 CRESS, Paris, France; 4 AP HP and Paris School of Economics, Paris, France; 5 Centre de Recherche des Cordeliers, INSERM, Sorbonne Université, USPC, Université Paris Descartes, Université Paris Diderot, Equipe d’accueil ETRES, F-75006 Paris, France; 6 Biomedicine Agency, Saint Denis, France; 7 UPEC, Creteil, France

**Keywords:** Organ transplantation, Teleimaging, Tele-diagnosis, Tele-expertise

## Abstract

**Background:**

The transplantation process usually takes place without transplant teams being able to use imaging data to assess graft quality. The decision of whether to go get the graft or not is therefore limited and suboptimal. “Cristal images” is a teleimaging project allowing real-time visualization of images of the organs of the donor. The objective of our study is to assess whether the use of a secure teleimaging can improve the effectiveness and efficiency of the procurement and transplantation processes.

**Methods:**

We will use the exhaustive national registry of organ allocation and transplantation, and compare outcomes before the deployment of “Cristal images” (years 2015–2016) and after it becomes operational (years 2018–2019) for heart, lung, liver and kidney transplant in a before-after study, combined with a preference elicitation study. The primary endpoint will be the number of successful organ transplantations. Secondary endpoints will be related to the efficiency of the transplant process (decision making, transportation, cost) and a preference elicitation study will determine the relative preferences of transplant teams towards few “Cristal images”’ components or potential developments, which are yet to be determined through a qualitative analysis based on interviews with professionals.

**Discussion:**

This study will provide stakeholders data on the efficiency of real-time visualization for transplant teams and identify the levers likely to influence the technology use among these teams.

**Trial registration:**

clinicaltrials.gov: NCT03201224, 13 June 2017, retrospectively registered.

## Background

Organ transplantation is the treatment of choice for patients with terminal organ failure. In France in 2017, organ transplant activity amounted to 5,729 transplants, an increase of nearly 30% since 2006 [1]. However, 16,413 patients remained on the national waiting list for transplantation as of January 1, 2018, and in 2017, 590 patients died while on the waiting list [1]. Improving organ procurement remains a challenge with the increasing age (almost 58 years) and associated co-morbidities of deceased donors resulting in “extended criteria” transplants [1].

The two major steps of the transplant process are 1) the evaluation by the donor procurement teams, and 2) proposal to the transplant teams, according to national allocation rules. Without the possibility of image transmission between the procurement and the transplant teams, decisions are made without any assessment of organ anatomy by the transplant teams. The transplant team accepting the graft will travel to the procurement site to verify the graft aspect. If the graft is ill-suited to their patient’s anatomy, the team has to relinquish the graft, which increases the risk of organ necrosis and loss due to prolonged ischemia.

Teleimaging has been shown to be feasible and reliable for pre-transplant decision support and procurement planning [2–6]. Teleimaging could therefore offer a solution to reduce the risk of non-procurement by allowing the transplant team to accept or not a graft after examining high quality images. “Cristal Images” is a teleimaging project funded by the French ministry of health to upload radiological images on local site to a server for remote visualization in streaming. Its objective is to make images of donor organs available to the transplant team early in the transplantation process, anonymously and securely.

## Methods/design

### Study design

We will evaluate “Cristal Images” by conducting a mixed-methods study involving an effectiveness study focusing on the processes and outcomes, and a preference elicitation study focusing on the perception of the transplant teams.

### Aim & setting

Our objective is to assess whether the use of a secure image transmission in the context of organ transplantation can improve the effectiveness and efficiency of the transplantation process and change the practice of the procurement and transplant teams.

### Effectiveness study

#### Participants and data source

Our data will be provided by an extraction of the national registry of transplantation. This registry is exhaustive, collects all relevant clinical data regarding the donor and the recipient, such as age, cause of death, organ status. It also collects process data, such as number of transplant teams contacted, time between organ procurement and transplantation.

#### Inclusion criteria

- Organs from a brain-dead donor among: kidney, liver, heart and lung.
- Donor age over 18
- Deceased in France

#### Non-inclusion criteria

- Organs of a patient deceased after a cardiac arrest (Maastricht Classification: Type II and III) [7]
- Organs of a patient deceased in French overseas territories

#### Design

We designed an uncontrolled before/after study. Ethically, it was not possible to use any prospective control group, as the underlying hypothesis for the deployment of teleimaging was that it could only benefit recipients and therefore equipoise was impossible if image transfer were made unavailable in some regions.

The “before” period ranged from 2015/01/01 to 2016/12/31 when the “Cristal Images” image transfer platform was unavailable, and the “after” period ranged from 2018/01/01 to 2019/12/31. The intermediate period will not be taken into account as it is a period of deployment, tests, debugging, and adoption of the new technology.

#### Endpoints

##### Primary endpoint

Graft survival rate, assessed clinically and biologically 3 months after surgery for kidney, and 1 year after surgery for liver, heart and lung.

##### Secondary endpoints

- Percentage of organs allocated but not collected
- Percentage of graft transportation without transplant
- Percentage of transplant team travel without organ collection
- Mortality or loss of graft function, 3 months after surgery for kidney and 1 year after transplantation for the other organs
- Number (Percentage) of extended criteria donors
- Cost of the transplantation process, by a microcosting approach, from the acceptance of the organ by phone by the medical transplant team until the transplantation.
- Proportion of image transmission by the transplant team during the procurement process

#### Statistical analyses

##### Sample size

In 2015, there were a total of 5746 transplants from deceased donors (heart: *n* = 471; lung: *n* = 345; liver: *n* = 1355; kidney: *n* = 2939). 335 (15.4%) vital organs (heart, lung, liver) proposed to transplant teams were finally not transplanted. 297 (10.1%) kidneys were collected but not transplanted.

Our hypothesis is that image transfer will reduce lost organs by 82 (11.6%) for vital organs (heart = 20, lung = 17, liver = 45) and about 100 (6%) for kidney.

With a probability of Type I Error of 0.05 and a Power a 90%, the sample size required is 1797 vital organs transplantations and 924 kidney transplantations, which is inferior to our estimated cohort size of deceased donors.

##### Type of analyses

All variables will be described globally and by group (donors included during the “before” versus “after” period). The description will include the numbers and percentages of the modalities for the qualitative variables and the minimum, maximum, mean, standard deviation and median for the quantitative variables.

The one-year overall mortality rate will be estimated for the two periods (before and after intervention). This will be modeled by a mixed-effects logistic regression allowing for the cluster (hospital) level as a random effect and the recipient as a fixed effect. The same type of analysis will be used for binary variables.

Quantitative variables will be modeled by mixed linear models.

The total duration of the decision-making process and the duration between the first proposal and the transplantation will be analyzed by a proportional-risk Cox model with random effects.

### Preference elicitation study

#### Participants and data source

Our data will be collected using questionnaires addressed to transplant teams. All the transplant teams working in hospitals authorized by the Regional Health Agencies (“ARS”) on the advice of the French Agency of Biomedicine (ABM) to perform kidney, liver, heart or lung transplant will be contacted for inclusion in the study, excepted the transplant teams working in pediatric hospitals or in hospitals located in French overseas departments, who will be excluded of the study to align with the main evaluation protocol. The questionnaires will include items about the preferences of the professionals regarding “Cristal Images” (utilization, components, evolutions) as well as questions to gather information on the activity of the respondents, such as their clinical specialty, the size of their hospital and team, the number of transplantations operated per year, their practice with “Cristal Images” etc.

#### Method

**Fig. 1:**
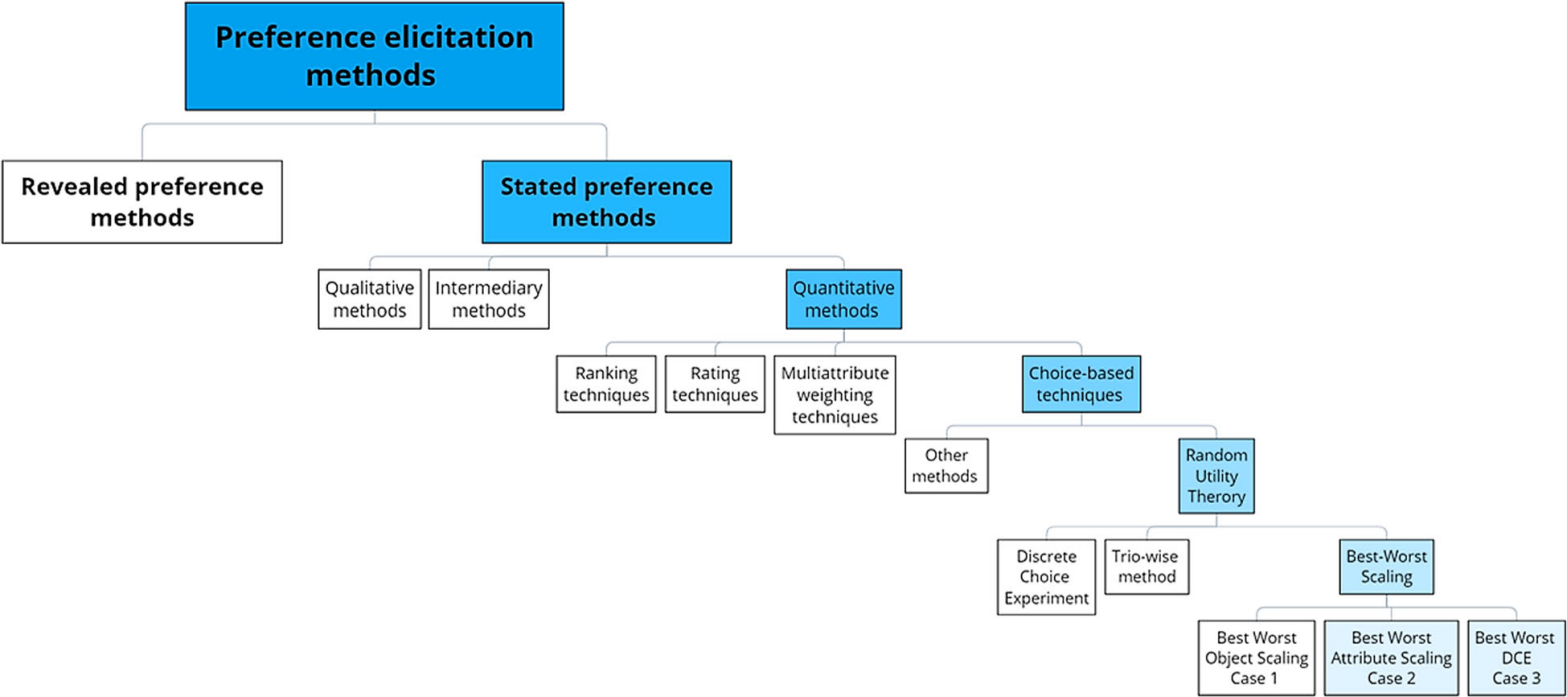
Classification of preference elicitation methods

An extensive review of the literature about preference elicitation methods was conducted beforehand, that allowed us to classify methods and to choose the Best Worst Scaling (BWS) methods, introduced by Finn and Louviere [8] and designed by Marley and Louviere [9], as the most relevant ones for our study (Fig. 1).

Revealed preference methods are based on the observation of actual utilization patterns; these methods are not applicable here, especially because our objective is to study how actual preferences relate to advisable evolutions of “Cristal Images”. Stated preference methods consist in asking respondents to choose between hypothetical situations in order to elicit their preferences. Among the different stated preference methods, we privileged quantitative methods because we can measure the relative strength of the different preferences and their relationships. Among them, the choice-based techniques are the closest to the decision-making process of professionals in their daily practices. From a research perspective, these methods imply a lower cognitive burden and a greater wealth of information. Methods based on the random utility theory have a strong theoretical basis, namely that an individual’s utility includes a systematic observable component and a random unobservable component [10]. Finally, among the random utility theory, Discrete Choice Experiment (DCE) and Best-Worst Scaling (BWS) have received extensive application in health in the 2000s–2010s, compared to the trio-wise method that is currently more cutting-edge. BWS optimizes the cognitive burden for respondents and the wealth of information; it also allows to directly compare attributes and finally, for Best-Worst DCE, to get a maximal amount of data for a given number of respondents. In sum, we pre-selected two BWS designs: Best-Worst Attribute Scaling and Best-Worst DCE.

#### Design

In a BWS design, the variable of interest is decomposed into attributes, each with different levels, and respondents are asked to identify their extreme preferences (best and worst items), among attribute levels within a single profile (Best-Worst Attribute Scaling) or among different profiles described by a number of attributes fixed at specific levels (Best-Worst DCE) [11, 12]. This enables comparison between the relative preferences for attributes and levels of a given object, namely the teletransmission of imaging in the transplantation process (“Cristal Images”) in this study.

Therefore, to design a study of preference elicitation based on the Best-Worst Attribute Scaling or the Best-Worst DCE methods, we need to determine the attributes and their levels corresponding to actual or potential characteristics of the intervention that transplant teams may value. To identify them, a qualitative study has been carried out from July 2018 to March 2019. Semi-directed phone interviews were conducted with professionals working in transplant teams included in the study. The sampling of this qualitative phase privileges diversity over representativity. Three to five interviews have been made per organ as well as per region (4 regions following the classification by the Regulation and Support Service (“SRA”) of the ABM), for a total of 16 interviews. The sampling also takes into account the number of transplantations performed per year by transplant teams as well as the role and the hierarchical position of interviewees. Interviews are transcribed for the purposes of the qualitative analysis to determine attributes and levels for the BWS design. The qualitative analysis will also guide the selection of the most appropriate design between Best-Worst Attribute Scaling and Best-Worst DCE.

## Discussion

This study was initially planned to start very early after the end of the technical implementation of “Cristal Images”. But technical implementation does not correlate well with professional adoption [13, 14], for reasons such as behavioral barriers, change management, resistance to change, social acceptability of teleimaging or organizational barriers, which emphasizes the necessity for teleimaging services to nicely fit into existing organizational structures.

The impact of telemedicine should be evaluated when it is routinely used by professionals [15]. However, there is no proper definition of the routine use. That means that we may need to modify the beginning of the period after intervention regarding the data that we will get about the “Cristal Images” uptake. However, as this study is funded by the French Ministry of Health, results are expected within 5 years, implying constraints on the time for the ‘after’ period.

Another potential issue for the evaluation is that we considered the transplantation process as a single process, regardless of the organ and the team. The procurement process differs between vital organs (heart, liver, lung) and kidney in the transplantation process: kidneys can be extracted by the local team and exported by courier service to the transplant team. Vital organs are ordinarily extracted by the transplant team. Moreover, depending on the organs, the teams do not require the same types of imaging, and there may be heterogeneity about availability of imaging (e.g. the full-body CT-scan, required by lung transplant teams, is often available, and cardiac ultrasound or coronary angiography are less available, because of technical issues or non-realization of the imaging). Therefore the utility of real-time visualization may vary with respect to organs and across transplant teams. This may have an impact on several endpoints and will be accounted for accordingly. This study will be a unique occasion to show such variations in the uptake and uses of the technology.

The preference elicitation study will generate evidence on the drivers and hurdles to an appropriate integration of the technology by transplant teams. It will also bring out the needs of these teams, current limitations of the teleimaging tool and prospects for further developments. Ultimately, we hope that professionals’ involvement in the preparatory phase will be instrumental to improve the uptake of transplant teams in the preference elicitation study.

Finally, the research, based on the mixed methods study, will provide stakeholders with data on efficiency of real-time visualization for transplant teams and identify the levers likely to influence the technology use among these teams.

## Data Availability

The datasets which will be analyzed during the current study will not be publicly available, as the French data protection law (Loi “Informatique et Libertés”) forbids to share data or to make it publicly available, even if they have been unidentified previously. Furthermore, the transfer of the data outside EU is not possible without getting a new specific authorization of the authority in charge of the enforcement of the “Informatique et Liberté” Law (named CNIL: https://www.cnil.fr/en/home). Therefore, an authorization must be granted by the CNIL before data can be made available; once this authorization obtained, data will be available upon request to DRCI-URC Eco (www.urc-eco.fr, kevin.zarca@aphp.fr).

## Abbreviations

ABM: Agency of Biomedicine
ARS: Regional Health Agency
BWS: Best-Worst Scaling
DCE: Discrete Choice Experiment
SRA: Regulation and Support Service
